# Potential of Carvacrol and Thymol in Reducing Biofilm Formation on Technical Surfaces

**DOI:** 10.3390/molecules26092723

**Published:** 2021-05-06

**Authors:** Maciej Walczak, Marta Michalska-Sionkowska, Daria Olkiewicz, Patrycja Tarnawska, Oliwia Warżyńska

**Affiliations:** Department of Environmental Microbiology and Biotechnology, Faculty of Biological and Veterinary Sciences, Nicolaus Copernicus University, Lwowska 1, 87-100 Torun, Poland; walczak@umk.pl (M.W.); mms@umk.pl (M.M.-S.); deo@doktorant.umk.pl (D.O.); oliwia.warzynska@vp.pl (O.W.)

**Keywords:** biofilm formation, microbial adhesion, carvacrol, thymol

## Abstract

Polyvinyl chloride (PVC), polypropylene (PP), polyethylene (PE), and stainless steel (SS) are commonly used in medicine and food production technologies. During contact with microorganisms on the surface of these materials, a microbial biofilm is formed. The biofilm structure is difficult to remove and promotes the development of pathogenic bacteria. For this reason, the inhibition of biofilm formation in medical and food production environments is very important. For this purpose, five naturally occurring compounds were used for antimicrobial screening tests. The two with the best antimicrobial properties were chosen to inhibit the biofilm formation of *Staphylococcus aureus* and *Pseudomonas aeruginosa*. After 3 days of exposure, thymol reduced the amount of biofilm of *Pseudomonas aeruginosa* within the range of 70–77% and 52–75% for *Staphylococcus aureus*. Carvacrol inhibited the formation of biofilms by up to 74–88% for *Pseudomonas aeruginosa* and up to 86–100% for *Staphylococcus aureus*. Those phenols decreased the enzyme activity of the biofilm by up to 40–100%. After 10 days of exposure to thymol, biofilm formation was reduced by 80–100% for *Pseudomonas aeruginosa* and by about 79–100% for *Staphylococcus aureus*. Carvacrol reduced the amount of biofilm by up to 91–100% for *Pseudomonas aeruginosa* and up to 95–100% for *Staphylococcus aureus*.

## 1. Introduction

Bacteria, in order to protect themselves from harmful environmental factors, are capable of creating multicellular structures generated by extracellular polymeric substances called biofilm [1,2,3]. This specific membrane allows bacteria to survive harsh conditions, such as extreme temperatures, high acidity and alkalinity [2], or great antibiotic concentrations [3]. Biofilm is also known for its ability to adhere to various surfaces, including food production equipment [3] and medical instruments [4,5,6]. The most commonly used polymers and materials in medicine are polypropylene (PP), polyethylene (PE), polyvinyl chloride (PVC), and stainless steel (SS). The formation of biofilm on medical materials is a serious problem [7] which causes about 80% of all medical infections [8]. The resistance on antimicrobial agents makes biofilms difficult to control, especially on medical equipment [9]. Biofilm adhesion depends on the physicochemical properties of medical materials, which are mostly their hydrophilicity and surface charge [10]. Both spoilage (*Pseudomonas aeruginosa*, *Enterococcus faecium*, *Micrococcus* spp.) and pathogenic microorganisms (e.g., *Staphylococcus aureus*, *Bacillus cereus*) can participate in the biofilm formation process [11]. Some of the biofilm formations involve potentially pathogenic strains (e.g., *Pseudomonas aeruginosa*, *Staphylococcus aureus*) that can lead us to difficult-to-treat infections [12,13,14]. *P. aeruginosa* strains are often the cause of opportunistic infections in cases of human chronic and immunosuppressive conditions [12,14]. Those infections can be difficult to treat because they are often associated with a high incidence of antibiotic resistance and biofilm formation [15]. *S. aureus* is a foodborne pathogen that can cause intoxications in humans by the consumption of contaminated food [16,17].

There are many methods to inhibit biofilm growth, including enzymatic degradation by bacteriophages; surface coating; physical methods; and natural compounds—e.g., essential oils [18,19,20]. Essential oils have been shown to exhibit antibacterial properties [20,21]. Compounds such as carvacrol, thymol, and eugenol are considered safe for people [22]. Carvacrol (2-methyl-5-(1-methylethyl) and thymol (2-isopropyl-5-methylphenol) have antibacterial, antifungal, and antiseptic activities [23,24].

Numerous studies are focused on the importance of biofilm, the potential infections it causes, and effective biofilm removal formed by different strains, including these pathogens: *S. aureus* and *P. aeruginosa*. The usage of essential oils or other natural substances in biofilm removal is also common [2,4,5,6,25,26,27,28]. However, our research focuses on the possibility of using natural substances to inhibit biofilm from different technical surfaces. Polypropylene, polyethylene, PVC, and stainless steel are current components of medical devices used in hospitals and balneological facilities. The surfaces of these materials play important roles in the initial microbial adhesion and biofilm formation. Aggressive disinfectants damage the surface of these materials and are odorous to staff and patients. Therefore, our goal was to investigate the possibility of limiting the development of biofilm by using other, more natural substances. These substances could be added to washing liquids.

**Figure 1:**
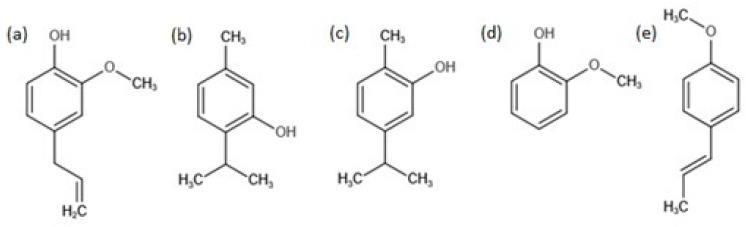
Chemical structure of eugenol (**a**), thymol (**b**), carvacrol (**c**), guaiacol (**d**), and trans-anethol (**e**).

The aim of the present study is to determine the inhibition effect of the natural compounds present in essential oils (i.e., eugenol (Figure 1a), thymol (Figure 1b), carvacrol (Figure 1c), guaiacol (Figure 1d), and trans-anethol (Figure 1e)) on the development of tested microorganisms, and, finally, to evaluate the effect of thymol (Figure 1b) and carvacrol (Figure 1c) on biofilm formation on technical/abiotic surfaces.

## 2. Results and Discussion

### 2.1. Screening Test

The results obtained from the screening test are shown in Table 1. Trans-anethol showed no inhibitory effect on the growth of all tested microorganisms. Trans-anethol naturally occurs in essential oils from fennel (*Foeniculum vulgare* L.) [29]. Roby et al. tested the antimicrobial properties of fennel essential oil (54% of trans-anethol in chemical composition) and their extracts. They discovered an inhibitory effect on the growth of *E. coli*, *S. typhi*, *B. cereus*, *S. aureus*, *A. flavus*, and *C. albicans*. However, Gram-positive bacteria were more sensitive compared to Gram-negative strains. *Foeniculum vulgare* Mill. (fennel) decoction (phenolic-enriched extract) was discovered to be a potential food preservative. The MIC values of *S. aureus*, *B. cereus*, *M. flavus*, *L. monocytogenes*, *P. aeruginosa*, *E. coli*, *E. cloacae*, and *S. typhimurium* were between 0.035 and 1.000 mg/mL. The antifungal activity of *Aspergillus* strains was 0.2–3.0 mg/mL [30]. Moreover, anethol is a major component of anise essential oil [31], and it has been shown that anise extracts inhibit the growth of both Gram-positive and Gram-negative bacteria strains [32]. However, our results were not similar to the results described in the literature, probably because the chemical composition of essential oil is a mixture of many different compounds, not only anethol.

Guaiacol is isolated from guaiac resin. It is the main constituent of creosote obtained from wood tar (beech). Guaiacol and its derivatives are antiseptics, gastric sedatives, flavorings, deodorants, fungicides, and parasiticides [33]. In our study, guaiacol also showed no inhibitory effect against tested strains. However, a small inhibition zone against *E. coli* was observed (concentration 4mg/disc).

Eugenol (4-allyl-2-methoxyphenol) is a naturally occurring phenol essential oil extracted from clove [34]. Eugenol in a concentration under 1 mg/disc was not effective against the tested strains. The growth of *E. coli* was gently inhibited by 1 mg/disc of eugenol. The highest concentration of eugenol (4 mg/disc) was effective against the tested microorganisms, except for *P. aeruginosa*.

The best results of the inhibitory effect were obtained after the use of thymol and carvacrol. The smallest dose of those phenols inhibited the growth of *S. aureus*. Thymol at the concentration of 0.1 mg/disc was also effective against *C. albicans*. Thymol at the concentration of 0.4 mg/disc was effective against *P. aeruginosa* and *E. coli*, while 1 mg/disc was necessary to inhibit the growth of *A. niger*. A 0.4 mg/disc concentration of carvacrol was already an effective dose against *E. coli* and *C. albicans*. To inhibit the growth of *P. aeruginosa* and *A. niger*, 1 mg/disc of carvacrol was needed. A less significant inhibitory activity of eugenol, compared to thymol and carvacrol, can be the result of its lower hydrophobicity. Therefore, a lower inhibition action may be associated with a weaker accumulation of this compound in the cell membrane of bacteria. In addition, the less intensive antimicrobial activity of eugenol may be due to the presence of the methoxy group in the ortho position, which impedes the release of the H + ion from the hydroxyl group of the compound [35,36]. A similar situation of the lower antimicrobial efficiency of eugenol compared to carvacrol was observed by Arfa et al. [35]. The authors suggest that the weaker antimicrobial activity of eugenol may be attributed to the lower hydrophobicity, where the octanol/water coefficient value (logP) was 2.73. The better antimicrobial effect showed compounds with logP larger than 3 (carvacrol 3.52, thymol 3.3) [35,37]. Engel et al. observed MIC values of 0.662 mg/mL for both thymol and carvacrol against *S. aureus* [38]. However, Lambert et al. showed that the MICs for thymol and carvacrol against *S. aureus* were 0.140 mg/mL and 0.175 mg/mL, respectively [39]. Different results were obtained by Miladi et al. (2017), where *S. aureus* was more susceptible to carvacrol than to thymol (MIC values: 64 µg/mL and 256 µg/mL, respectively) [22]. Moreover, fluconazole-resistant *Candida* isolates were sensitive to thymol and carvacrol, where carvacrol showed a greater sensitivity index. Lipophilic compounds probably penetrate into the cells and target the ergosterol biosynthesis pathway, thus impairing its biosynthesis [40]. After screening tests were performed, the authors decided to choose only thymol and carvacrol for further investigation.

### 2.2. Total Biofilm Amount

**Figure 2:**
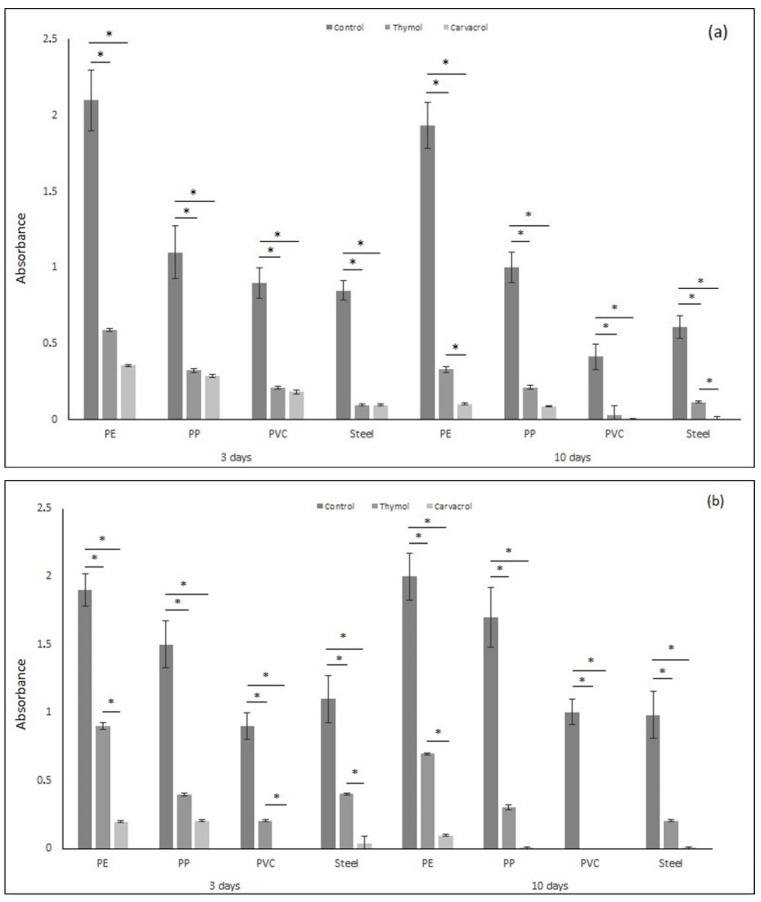
The total amount of biofilm (based on absorbance) formed by (**a**) *Pseudomonas aeruginosa* and (**b**) *Staphylococcus aureus* on materials (PE, PP, PVC, and stainless steel) after applying tested substances (thymol and carvacrol) according to the time of exposure. All analyses were performed in triplicate. Data are presented as mean ± SD (one-way ANOVA test and Tukey test were performed to determine the statistical significance between the indicated groups (* *p* < 0.05)).

The results of the total biofilm amounts are presented in Figure 2. *Pseudomonas aeruginosa* and *Staphylococcus aureus* form biofilm on surfaces of PE, PP, PVC, and SS materials. The biofilm amount on PVC and steel surfaces was smaller compared to the biofilm amount created on PE and PP surfaces for both *P. aeruginosa* and *S. aureus* for 3 and 10 days of exposure. Thymol and carvacrol reduced the amount of biofilm formed on each tested surface in the short and long time periods of exposure compared to the control samples.

Thymol reduced the biofilm formation of *P. aeruginosa* in the range of 70–77% after 3 days and ca. 80% after 10 days for PE, PP, and SS materials compared to the control samples. The biofilm amount was reduced by 100% 10 days after thymol application against *P. aeruginosa* for PVC sample. Thymol induced the inhibition of biofilm formation against *S. aureus* by up to 52–75% acting in a short time, and by up to 79–100% after a long time exposure.

Carvacrol reduced the amount of biofilm on each tested material. Compared to the results obtained for thymol, carvacrol was more efficient and the amount of biofilm was much smaller. After 3 days of exposure, carvacrol inhibited the formation of biofilms by up to 74–88% against *P. aeruginosa* and by up to 86–100% against *S. aureus*. After 10 days of exposure, the reduction in biofilm amount was higher, reaching up to 91–100% against *P. aeruginosa* and 95 to 100% against *S. aureus*. Moreover, PVC and SS materials were more resistant to biofilm formation compared to PE and PP.

Upadhyay et al. (2013) showed that thymol and carvacrol effectively inhibit biofilm formation and inactivate mature biofilms of *Listeria monocytogenes* on polystyrene and stainless steel [41]. Other in vitro studies showed that thymol can kill and significantly reduce *Actinobacillus pleuropneumoniae* biofilm formation by destroying the cell membrane structure, which results in the leakage of the cellular contents [42]. De Oliveira et al. (2017) discovered that the *Thymus vulgaris* L. extract promoted the control of biofilms of interest to oral health. The main terpene components of *T. vulgaris* extract are thymol, carvacrol, p-cymene, γ-terpinene, caryophyllene, linalool, and borneol. The authors discovered that 5 min of exposure to *T. vulgaris* L. extract at the concentration of 200 mg/mL reduced the amount of monomicrobial biofilms built by *C. albicans, S. aureus, E. faecalis, S. mutans,* and *P. aeruginosa,* as well as polymicrobial biofilms [43]. Moreover, essential oil components such as eugenol, thymol, and thymoquinone may show a synergistic effect as an antimicrobial agent against *S. epidermidis,* as well as its biofilm development [44]. Furthermore, it has been shown that collagen materials enriched with thymol may reduce *S. aureus* biofilm formation [45]. The effect of biofilm reduction may not necessarily be connected to the biocidal effect. It was shown that carvacrol may inhibit biofilm formation without reducing cell viability [46]. Carvacrol probably influences the gene coding of quorum sensing [47]. Therefore, those phenols may potentially be used as environmentally friendly compounds to reduce contaminations and infections [41].

### 2.3. Biofilm Hydrolytic Activity

**Figure 3:**
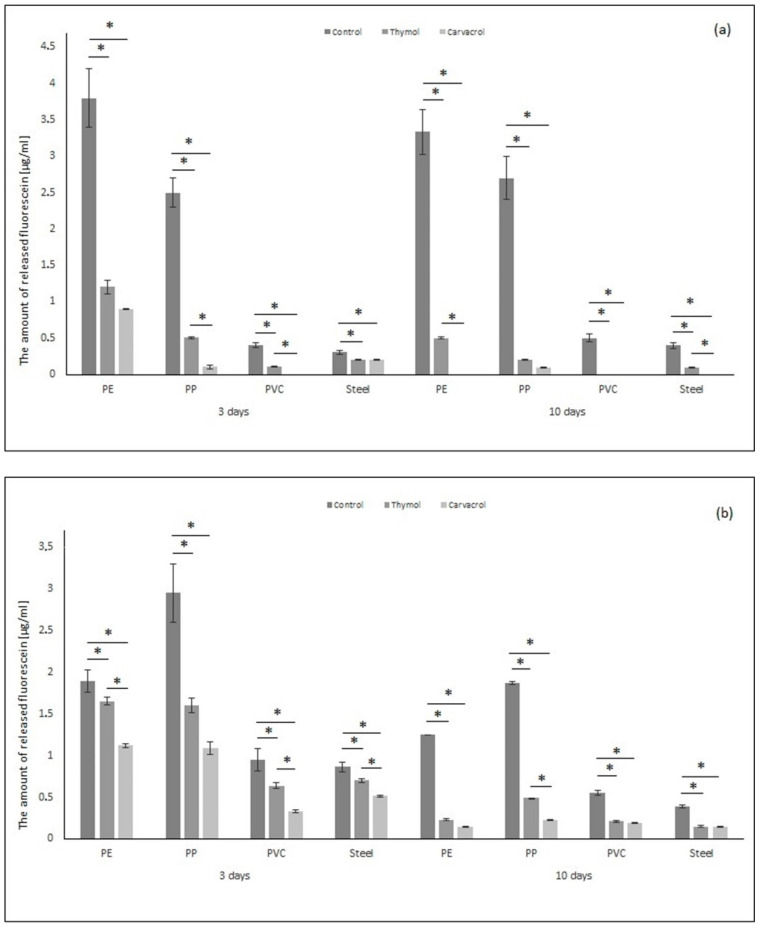
Hydrolytic activity of biofilm formed by (**a**) *Pseudomonas aeruginosa* and (**b**) *Staphylococcus aureus* on tested materials (PE, PP, PVC, stainless steel), after application of thymol and carvacrol depending on the time of exposure. All analyses were performed in triplicate. Data are presented as mean ± SD (one-way ANOVA test and Tukey test were performed to determine the statistical significance between the indicated groups (* *p* < 0.05)).

Fluorescein diacetate (FDA) hydrolysis is widely accepted as an accurate and simple method for measuring total microbial activity. Colorless fluorescein diacetate is hydrolyzed by both free and membrane bound enzymes, releasing a colored end product, fluorescein, which can be measured by spectrophotometry [48]. Figure 3a shows the amount of released fluorescein by biofilm built by *P. aeruginosa* (µg/mL). The amount of released fluorescein is smaller after thymol and carvacrol application. The hydrolytic activity of biofilm formed by *P. aeruginosa* after 3 days on the PE sample was 68% and 76% smaller after thymol and carvacrol were used, respectively. After 10 days, thymol reduced the amount of fluorescein by 85%. Carvacrol reduced the amount of released fluorescein by 100%. The amount of released fluorescein in the PP sample was 80% and 93% lower after thymol use (after 3 and 10 days, respectively) and 96% lower after carvacrol application compared to the control. The hydrolytic activity for PVC and SS samples was lower compared to PE and PP. Additionally, carvacrol reduced the amount of released fluorescein by 100% for the PVC sample in the short time of exposure and for PVC and SS in the long time of exposure, as well as after thymol use for the PVC sample after 10 days of exposure.

Figure 3b shows the results of the amount of released fluorescein by *S. aureus* enzymes. The total esterase activity was smaller after thymol and carvacrol application compared to control samples. The reduction in the amount of fluorescein after thymol use was up to 12–45% (3 days of exposure) and up to 52–82% (10 days of exposure). The application of carvacrol reduced the amount of fluorescein by up to 40–65% (3 days of exposure) and up to 61–88% (10 days of exposure). The biggest reduction in the amount of released fluorescein may be observed for PP sample after phenols application in both times. It can be observed that carvacrol decreases the activity of the esterase of *S. aureus* and *P. aeruginosa* to a greater extent than thymol. Moreover, longer-term phenol exposure causes lower microbial activity. Boonruang et al. (2017) showed that the material may be enriched with monoterpene. They prepared polymeric material made of poly (lactic acid) with incorporated thymol and R-(-)-carvon inside the material. This resulted in a receipt of packaging material potentially used in controlling postharvest diseases in fresh food produce [49].

### 2.4. Live and Dead Staining

**Figure 4:**
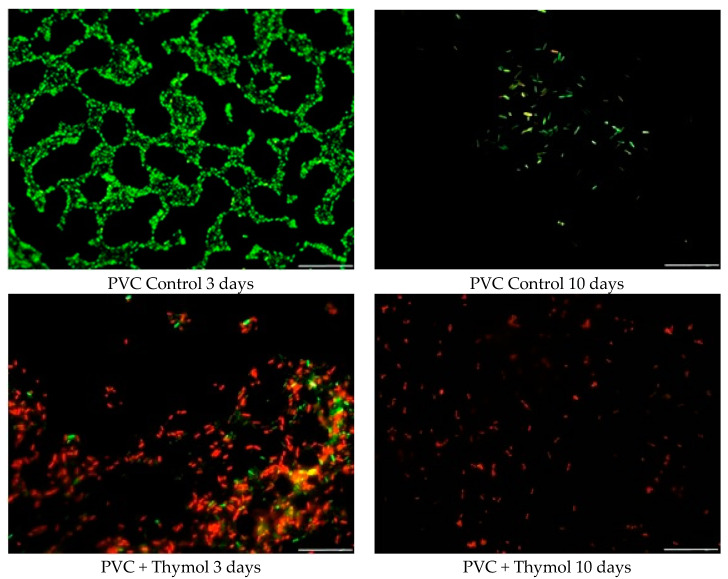
Changes in the quantity and viability of *Pseudomonas aeruginosa* biofilm on the PVC surface, before and after thymol addition.

Figure 4 shows sample pictures of the visual biofilm amount, distribution, and physiological state of *Pseudomonas aeruginosa* after 3 and 10 days of biofilm formation on PVC material. In the control sample, after 3 days of biofilm formation the initial stages of biofilm development can be observed. Most of the bacteria were stained green, meaning that there were living cells. Moreover, rosettes built of living cells were observed. After 10 days, the amount of biofilm significantly decreased.

After 3 days of biofilm development on the PVC sample with thymol addition, both live (green) and dead (red) cells can be observed, however, the number of dead cells was higher compared to that of live cells. Moreover, the number of total cells increased compared to the control sample. This was probably caused by the sedimentation of dead cells on the PVC surface after thymol action. After 10 days, no biofilm was observed on the PVC sample with thymol addition. It is due to this effect that dead cells were autolyzed after 3 days, and the new cells could not proliferate because of the thymol presence. This resulted in the single dead cells seen on the PVC surface.

Thymol and carvacrol successfully inhibit the growth of tested bacteria strains, molds, and yeasts. Moreover, thymol and carvacrol inhibit the biofilm formation of *S. aureus* and *P. aeruginosa*. Those phenols also reduce the hydrolytic activity of pathogenic bacteria. The results suggest that thymol and carvacrol may be successfully used as antimicrobial and antibiofilm agents for materials commonly used in the industry and play a positive role in the increase in hygiene level. As naturally occurring compounds, they are part of the assumptions of green chemistry and are not harmful to the environment. It should also be emphasized that studies with carvacrol and thymol prove their non-toxicity towards human cells and even their protective properties against harmful substances [50,51,52], which makes them safe for use by humans.

## 3. Materials and Methods

### 3.1. Bacterial Strains Used in the Research

The micro-organisms Escherichia coli ATCC8739, Pseudomonas aeruginosa, ATCC15442, *Staphylococcus aureus* ATCC6538, *Candida albicans* ATCC10231, and *Aspergillus niger* ATCC16404 were used for the study.

### 3.2. Tested Materials Types

Polyvinyl chloride (PVC), polypropylene (PP), polyethylene (PE), and stainless steel (SS) were used as the tested materials. Their characterization is presented in Table 2.

### 3.3. Screening Test

Thymol (Roth), carvacrol, eugenol, guaiacol, and trans-anethol (Sigma-Aldrich) were chosen as antimicrobial agents for screening tests. Substances which demonstrated the best results were chosen for the following studies.

Several 2% and 0.2% solutions of thymol, carvacrol, eugenol, and trans-anethol were prepared using 96% ethanol as a solvent.

Whatman No. 3 paper discs (1 cm diameter) were sterilized in an autoclave (117 °C, 20 min). Each prepared solution was applied to the discs in the amounts of 50 and 200 µL, and dried in a laminar flow chamber to evaporate the solvent. The final amounts of tested compounds applied to the discs were 0.1, 0.4, 1, and 4 mg.

The bacteria were cultured in flasks containing 50 mL of medium composed of (g L^−1^): bacteriological peptone (5) and yeast extract (3), pH = 6.8–7.2. Molds and yeasts were cultured on Malt Extract Agar (Biocorp) medium, pH = 5.5–5.8. After cultivation, the bacterial cultures were diluted with saline salt to obtain the optical density of 0.5 in McFarland. The biomass of *A. niger* was mechanically removed from the agar plate using saline salt and cells spreader. Then, the suspension was diluted with sterile saline solution to obtain the optical density of 0.5 in McFarland. Petri plates containing 20 mL of Mueller-Hinton culture medium (for bacteria) and Malt Extract Agar (Biocorp) medium (for yeast and molds) were inoculated with 100 μL of microbial suspension. Paper discs containing different amounts of the tested compounds were placed on the surface of the medium with microorganisms. The plates were placed in a refrigerator for 2 h to allow the diffusion of the compounds to the medium and then were incubated at 37 °C for 24 h (26 °C for 48 h for molds). The antimicrobial activity was evaluated by measuring the inhibition zone (mm) against the tested microorganisms [45].

### 3.4. Biofilm Formation on PVC, PE, PP and SS

*Staphylococcus aureus* and *Pseudomonas aeruginosa* were used as a model of biofilm-forming bacteria. Erlenmeyer flasks (capacity of 100 mL), each containing 50 mL of nutrient broth (composition [g L^−1^]: peptone: 5.00; yeast extract: 3.00), were sterilized in an autoclave for 20 min. Sterile medium in the flasks was inoculated with 0.1 mL of cell suspension (OD = 0.5) of *S. aureus* or *P. aeruginosa*. PVC, PE, PP, and SS sheets were cut into plates of 1 × 1.5 cm sterilized overnight in 70% propanol. Plates of different materials sterilized and rinsed in sterile water were added to the inoculated flasks. The 300 µL of thymol and carvacrol solutions in ethanol were added to experimental flasks. The total concentration of thymol and carvacrol was 0.5 mg/mL, ethanol in the same volume was added to the control flasks. The flasks were incubated at 37 °C for 3 and 10 days and were kept under observation for biofilm formation. At each time point, some plates of each material were taken out from each experimental and control group and were tested for their amount of biofilm estimation.

### 3.5. Estimation of Total Biofilm Amount

Biofilm assay was specified using the method described by Dieser et al. (2017) with minor changes [53]. Every material plate was placed in each well of a 24-well microtiter plate. Next, 2 mL of 1% (*w/v*) crystal violet (Sigma-Aldrich) solution was added to each well and the plate was incubated for 15 min at room temperature. Subsequently, the biofilm was fixed by drying (1 h, 60 °C). The plates were then washed, the dye was solubilized in 0.95% ethanol, and the absorbance at 570 nm was determined.

### 3.6. Estimation of Biofilm Hydrolytic Activity

After the incubation of PVC, PE, PP, and SS sheets in flasks with bacterial cultures (as above), the plates were taken out and rinsed thrice with PBS buffer to remove any unbound bacteria. As the next step, the material plates were transferred to a new sterile microtiter plate for the assessment of the hydrolytic activity of biofilm. After each well was filled with 2 mL of sterile PBS buffer, 10 µL of fluorescein diacetate (FDA) (Sigma-Aldrich) was added to the wells [6]. The samples were incubated in the dark for three hours at 37 °C. The amount of fluorescein released (the result of the hydrolytic activity of extracellular enzymes) was measured using a spectrofluorometer Hitachi F-2500, at the excitation wavelength of 480 nm and the emission wavelength of 505 nm. Finally, the results were expressed as the mean of three replicates.

### 3.7. Live and Dead Staining

For the visual observation of the distribution of live and dead bacteria in biofilm, LIVE/DEAD BacLight (Invitrogen Molecular Probes, Eugene, OR, USA) was used. SYTO9, which stains live bacteria with green fluorescence, and propidium iodide, which stains dead bacteria with red fluorescence, were mixed 1:1 and combined with PBS by adding 3 μL of the mixture per 1 mL of PBS to prepare a stain solution. The plates of the investigated materials were placed in the stain solution and reacted for 15 min. The stained biofilm or cells were observed under an epifluorescence microscope (Nikon, Eclipse 200).

## Figures and Tables

**Table 1:** Antimicrobial activity of tested compounds using the disc diffusion assay.

Inhibition Zone Diameter [mm]																				
	Thymol				Carvacrol				Eugenol				Guaiacol				Anethol			
mg/disc	0.1	0.4	1	4	0.1	0.4	1	4	0.1	0.4	1	4	0.1	0.4	1	4	0.1	0.4	1	4
*S. aureus*	11	15	17	45	15	29	39	45	0	0	0	16	0	0	0	0	0	0	0	0
*P. aeruginosa*	0	11	12	12	0	0	11	11	0	0	0	0	0	0	0	0	0	0	0	0
*E. coli*	0	12	18	25	0	12	28	32	0	0	11	21	0	0	0	11	0	0	0	0
*C. albicans*	11	13	18	36	0	12	30	37	0	0	0	23	0	0	0	0	0	0	0	0
*A. niger*	0	0	11	14	0	0	11	18	0	0	0	11	0	0	0	0	0	0	0	0

**Table 2:** The characterization of the used materials.

Material	Type	Roughness Ra [mm]	Thickness [mm]
PP	H	0.001	5
PE	500	0.002	5
PVC	U	0.003	5
SS	304	0.002	0.2

## Data Availability

The data presented in this study are available on request from the corresponding author.

## Sample Availability

Not available.
